# Dynamically adjusted strategy in response to developments in the COVID-19 pandemic as a new normal

**DOI:** 10.1186/s12992-021-00746-9

**Published:** 2021-08-09

**Authors:** Weifeng Shen

**Affiliations:** Department of Emergency Medicine, the Second Affiliated Hospital, Zhejiang University School of Medicine, Institute of Emergency Medicine, Zhejiang University, China. 88# Jiefang road, Shangcheng District, Hangzhou, China

**Keywords:** COVID-19, Pandemic, Response, Strategy, Public health

## Abstract

Presently, the developments of COVID-19 situation in different countries and regions have clearly differentiated. Due to differences in resources, infrastructure, and awareness of epidemic prevention and control, capabilities for COVID-19 prevention and control in various regions have also shown a significant imbalance as the COVID-19 epidemic is entering a new normal. The objectives of this study are to provide dynamically adjusted strategies in response to developments in the COVID-19 pandemic as a new normal. In the face of the new normal, one key is normalizing epidemic prevention and control. As part of this, we should implement precise policies based on the dynamics of the COVID-19 epidemic and particular response needs. In ongoing COVID-19 prevention and control, we must pay attention to new vulnerabilities and new features in the dynamics of the epidemic. In this study, health and government officials can benefit from insights of preparing ourselves for long-term challenges and both certainties and uncertainties in a future facing COVID-19.

## Background

At present, developing public health measures, drugs and vaccines to respond to the Coronavirus disease 2019 (COVID-19) pandemic has become a global effort [1, 2]. The characteristics of the COVID-19 pandemic have changed [3–5]. The dynamics of the COVID-19 pandemic in different countries and regions have shown obvious differentiation. The developments of COVID-19 situation are more reflected in the occurrence and spread of new variants, which can be impacted by the nature of COVID-19, human activities and containment measures. Different situations have arisen in different regions and populations [6, 7], such as a spreading of the epidemic, weakening of the epidemic, fundamentally controlled situation, and cases of epidemic rebound. The risk levels of the epidemic in different regions vary and there is a risk of the epidemic spreading across regions [8, 9]. With the gradual relaxation of restrictions, it will be more difficult to control the spread of COVID-19 across regions. Due to differences in resources, infrastructure, and awareness in prevention and control, capabilities for COVID-19 prevention and control have shown significant imbalance in various regions. The current COVID-19 pandemic is now entering a new normal. Under this new normal, how to effectively and accurately prevent and control the COVID-19 pandemic is a very important issue. More precise strategies need to be implemented in accordance with the dynamics of the COVID-19 pandemic and requirements for epidemic prevention and control. The main objectives of this study are to provide dynamically adjusted strategies in response to developments in the COVID-19 pandemic as a new normal.

## Discussion

### Promoting vaccination

Currently, the COVID-19 vaccination is being actively promoted, the global COVID-19 pandemic has significantly shown signs of control, but there are still signs of a new wave in some countries and regions [10]. Current situation shows that in the process of widely promoting vaccination, it is still necessary to form the overall response capacity of COVID-19. Otherwise, in some areas where the COVID-19 pandemic develops rapidly and vaccination is relatively slow, the progress of epidemic may be faster than vaccination. There is no doubt that vaccination is one of the most important epidemic prevention and control measures, which needs to be carried out in an orderly manner. In areas with serious epidemic and epidemic rebound, vaccination should be accelerated to form an immune barrier of population. Vaccines are considered a key resource for COVID-19 prevention and control. Development capabilities, production capabilities, cold-chain transportation capabilities, and distribution strategies for vaccines are a critical capability for the control of COVID-19.

### Long-term trends of COVID-19 and regular epidemic prevention and control

The second wave or long-term course of the COVID-19 pandemic presents an important challenge that needs to be faced [11, 12]. Studies have shown that severe acute respiratory syndrome coronavirus 2(SARS-CoV-2) can survive and spread even in a humid and high temperature environment, and its transmissibility is not weakened [13]. At its current stage, COVID-19 prevention and control is not only limited to an emergency response, but also encompasses continuously constructing needed infrastructure and consolidating basic systems of normalized epidemic prevention and control. It aims to improve the capabilities of real-time monitoring, risk-assessment, scientific decision-making, the implementation of public health measures, and public education [14]. For severely affected areas, emergency measures to curb the spread of COVID-19 are given a higher priority. For areas where the epidemic is under control, it is better to focus on infrastructure for epidemic prevention and control, and improve basic capabilities for the prevention and control of epidemic rebounds in the region as well as address imported COVID-19 cases across regions. For long-term trends of the COVID-19 epidemic, the key is to focus on preparing supplies, equipment, venues, and personnel to ensure the sustainability of epidemic prevention and control systems.

### Quickly identifying and curbing a COVID-19 epidemic rebound

At present, there are still many unknowns and uncertainties about COVID-19. There is the risk of a rebound of the COVID-19 epidemic. Due to the high infectivity and asymptomatic spread of SARS-CoV-2 [15], capabilities for early identification, early warning, and rapid response to COVID-19 still require continuous improvement. Even after the epidemic is fully controlled, clusters of COVID-19 may still occur in localized areas [16]. Through early detection and isolation of newly-confirmed COVID-19 cases; careful epidemiological investigations; accurate identification of new points of COVID-19 outbreaks; timely identification of epidemic transmission chains; and precise implementation of contact tracing, isolation, and quarantine; new outbreaks of COVID-19 can be successfully contained. In coming months and years, COVID-19 outbreaks, similar to those already experienced, may occur possibly in multiple areas at the same time. This is the new normal. For this reason, it is necessary to continuously improve capabilities for precise and timely identification and rapid response for multi-point triggers of epidemic outbreaks. Three core capabilities include: the capability to quickly trace contacts, such as by using digital technologies for contact tracing [17]; the capability to rapidly provide nucleic acid detection [18], such as through large-scale nucleic acid detection for entire regions in a short time; and finally, the capacity for the admission and treatment of critically ill patients [19], such as ICU capacity and health capabilities in a particular area. The range of nucleic acid detection for screening should be determined by the identification of contacts and by focal points found in epidemiological COVID-19 investigations. If the latter two are not clear, the screening range of nucleic acid detection should be moderately expanded. Large-scale nucleic acid testing can be used to quickly screen patients who have been infected with SARS-CoV-2 from a particular targeted population [20]. For key populations which need screening, nucleic acid tests can be performed multiple times according to prevention and control requirements. The risk factors for local COVID-19 epidemic rebounds may come from within or from outside a region. Global epidemic prevention and control is a holistic process [21]. To reduce the risk of cross-border or cross-regional transmission of COVID-19, countries and regions with high cross-border or cross-regional risks of COVID-19 transmission should cooperate by establishing a joint prevention and control mechanism for COVID-19. Effective communication plays an important role in the prevention and control of COVID-19. It is particularly important in COVID-19 data sharing [22, 23]. Even in phases of gradually lifting restrictions imposed due to COVID-19, regional coordination is also needed. Studies have shown that a coordinated COVID-19 exit strategy can help avoid a second wave of COVID-19 outbreaks in Europe [24].

### Long-term dynamic monitoring of SARS-CoV-2 transmission and mutation

The interpersonal transmission of SARS-CoV-2 and changes in pathogenicity are continuously monitored. Recently, it has been reported that newborns have been infected with SARS-CoV-2 from their mothers [25], a finding which still needs further supporting evidence. There is uncertainty about the potential spread of SARS-CoV-2 across species, and there is a continued focus on the issue in the normalization of epidemic prevention and control. It is essential to monitor the susceptibility of animals to SARS-CoV-2 and their infectivity to similar species and humans [26]. Although COVID-19 has not been classified as a zoonotic disease, from the perspective of the normalization of epidemic prevention and control, a “one health” strategy still must be followed [27]. An essential issue related to the spread of SARS-CoV-2 is that the risk of SARS-CoV-2 mutation continues [28]. The SARS-CoV-2 mutation is consistent with trends of the COVID-19 epidemic and is of concern in the preparation of related public health measures. The SARS-CoV-2 virus mutates to create new variants. It is necessary to dig out the key mutations by genomic surveillance of the virus [29, 30]. Laboratory studies have shown that the D614G mutation of SARS-CoV-2 may lead to accelerated virus replication [31]. Whether this mutation increases the infectivity of SARS-CoV-2 in the actual environment will require continuous monitoring. Vaccine development also requires long-term monitoring of SARS-CoV-2 mutations.

### Long-term dynamic preparation for the interaction of SARS-CoV-2 with natural and social factors

Natural and social factors will have an important impact on the development of the COVID-19 epidemic. In terms of natural factors, the impacts of environmental temperatures on the spread of SARS-CoV-2 have been preliminarily studied [13]. There are more natural factors affecting COVID-19 that warrant further study. The impact of extreme meteorological disasters on the spread of SARS-CoV-2 has not yet been reported. The combined impact of COVID-19 and natural disasters may increase the severity of harm to humans. A series of studies have been carried out on the impact of social factors such as poverty [32], social distancing policies [33] and income gap factors [34] on the epidemic. Evidence shows that exposure to air pollution might be an additional factor influencing COVID-19 morbidity and mortality rates [35]. Further, COVID-19 will also have a profound impact on the natural environment and society as a whole. Studies have shown that COVID-19 lockdowns may change the way the earth moves [36]. COVID-19 lockdown measures cause changes in human activities, which may cause global quieting of high-frequency seismic noise [37]. The most direct interactive impact is that the COVID-19 pandemic has caused the medical service system to function at full capacity or has even overloaded it, which poses a challenge for health systems to meet the needs of epidemic prevention and control as well as daily medical needs. For instance, the global pandemic of COVID-19 can, in some areas, interrupt the continuity of medical services for AIDS, tuberculosis, malaria, and tumors [38, 39]; delay diagnosis for some cancer patients [40]; and will lead to a decline in global fertility [41]. It will also have profound effects on children such as lower academic achievement, poverty, and hunger [42]. These effects are developing along with the COVID-19 epidemic itself. If there is insufficient preparation or an improper response, the impact of COVID-19 may become even more serious than the epidemic itself.

### The priority to focus on vulnerable populations and links with epidemic prevention and control

During the COVID-19 pandemic, the overall effect of epidemic prevention and control depends strongly on the impact on the weakest and most vulnerable parts of society. People as a whole are generally susceptible to SARS-CoV-2, but people with chronic underlying diseases may see an increased risk of severe COVID-19 infection [43]. This is an issue that needs particular attention. As the shortcomings of epidemic prevention and control are examined, rapid detection capabilities for SARS-CoV-2 must be mentioned. One of the important reasons for large-scale outbreaks of COVID-19 in localized areas is that the SARS-CoV-2 nucleic acid detection capabilities cannot keep up with the surge in the population that needs to be tested. This shortcoming of in curbing the epidemic should be addressed as a priority. Low-income countries and regions will face serious challenges in epidemic prevention and control, and require humanitarian assistance and medical resources for life-saving and preventative measures [44]. One of the needs of epidemic prevention and control is that free nucleic acid test should be carried out for poorer populations needing screening. For economically vulnerable social groups, epidemic-related care costs should be provided in order to allow temporary healthcare security. Vulnerable groups and vulnerable links in prevention and control may exist for a long time, or they may change following active support. It is also possible that new vulnerable groups or new vulnerable links in prevention and control will be discovered. Although low-income countries have obvious vulnerabilities in epidemic prevention and control due to their poor health infrastructure, as long as effective measures of COVID-19 are taken, these vulnerabilities can be significantly reduced [45]. Enough attention should be paid to the provision of mental health services for those infected or people whose loved ones contracted COVID-19 [46, 47].

### The new risk prevention and control of the spread of COVID-19 under the new normal

Recently, the issue of whether SARS-CoV-2 is spread through wet markets, live poultry trading markets, or through food from high-risk areas has attracted widespread attention. According to reports [48], SARS-CoV-2 on the outer packaging of imported cold chain-products has been detected. Such occurrences should be considered as new risk factors and effective preventive measures should be taken. In the evolution of the COVID-19 epidemic, new risk factors that need to be studied may continue to increase. It is necessary to neither under-react nor over-react to potential new risk factors in order to carefully confirm or eliminate those potentially related to the COVID-19 epidemic. This is also the new normal of current epidemic prevention and control.

### Implementing COVID-19 prevention and control measures based on risk levels, actual needs and the effects of epidemic prevention and control

The reported data and survey collection data for COVID-19 form an important basis for decision-making. However, not all of such data is sufficiently considered in the dynamic course of the COVID-19 epidemic, and epidemic prevention and control has difficulty keeping up with the rapid development of the COVID-19 epidemic. Moreover, some theoretically effective prevention and control measures may not show their effects under specific environments or at specific phases of the COVID-19 epidemic. Therefore, strategies for COVID-19 prevention and control should be based on the integration of the data obtained, risk judgment, and the effects of epidemic prevention and control. Public health interventions, such as city closures and traffic restrictions, played an important role in blocking the rapid spread of COVID-19 in early stages [49]. In the next stages of the COVID-19 epidemic, a prevention and control strategy of division and classification should be implemented according to the actual needs, risk levels, and effects of epidemic prevention and control. For areas where the COVID-19 pandemic is still on the rise, strict closed measures should continue to be implemented [50]. For areas where COVID-19 is largely under control, both epidemic prevention and control and the resumption of work should be taken into account-while utilizing the experience gained in the previous stage [51]. With the gradual accumulation of experience in epidemic prevention and control and the continuous improvement of the application of advanced information technologies, the risk classification of the epidemic in key epidemic areas will be more detailed and precise. Individuals wearing surgical masks, social distancing, and training to enhance human immunity can effectively block SARS-CoV-2 infection [52–54]. These public health measures can be adopted even over the long term and even in areas of low-risk for COVID-19 until the epidemic is over [55, 56]. The public’s compliance with these measures will affect the prevention and control effects. In the process of lifting restrictions after the COVID-19 epidemic has subsided, a reverse division and classification approach can also be adopted in the gradual lifting of restrictions. When some regions show signs of a severe rebound of COVID-19, closure policies can still be reinstated.

### Taking account of COVID-19 prevention and control in the daily medical order, preparation, and response to other emergencies is the new normal

During the global pandemic of COVID-19, no country or region can remain completely unconnected or disregard its impact - all must actively curb the spread of COVID-19. As COVID-19 may persist for a long time, effective ways of minimizing the impact of COVID-19 on daily medical operations and ensuring daily medical capacity must be adopted. This requires added work time from medical staff compared to the usual. More innovations in medical services, such as internet medical services, can be used to divert daily medical needs [57]. More importantly, establishing a medical resource allocation mechanism to reasonably balance the special medical needs of COVID-19 prevention and control with the daily demands of medical care is a key step. In addition, some regions may face more severe challenges than others in managing the massive stress of daily medical needs and special medical needs for COVID-19 prevention and control when also faced with a surge in medical needs caused by other potential emergencies. In order to cope with such possible complications, it is necessary to establish an emergency mechanism for multi-line operations.

## Conclusions

**Fig. 1:**
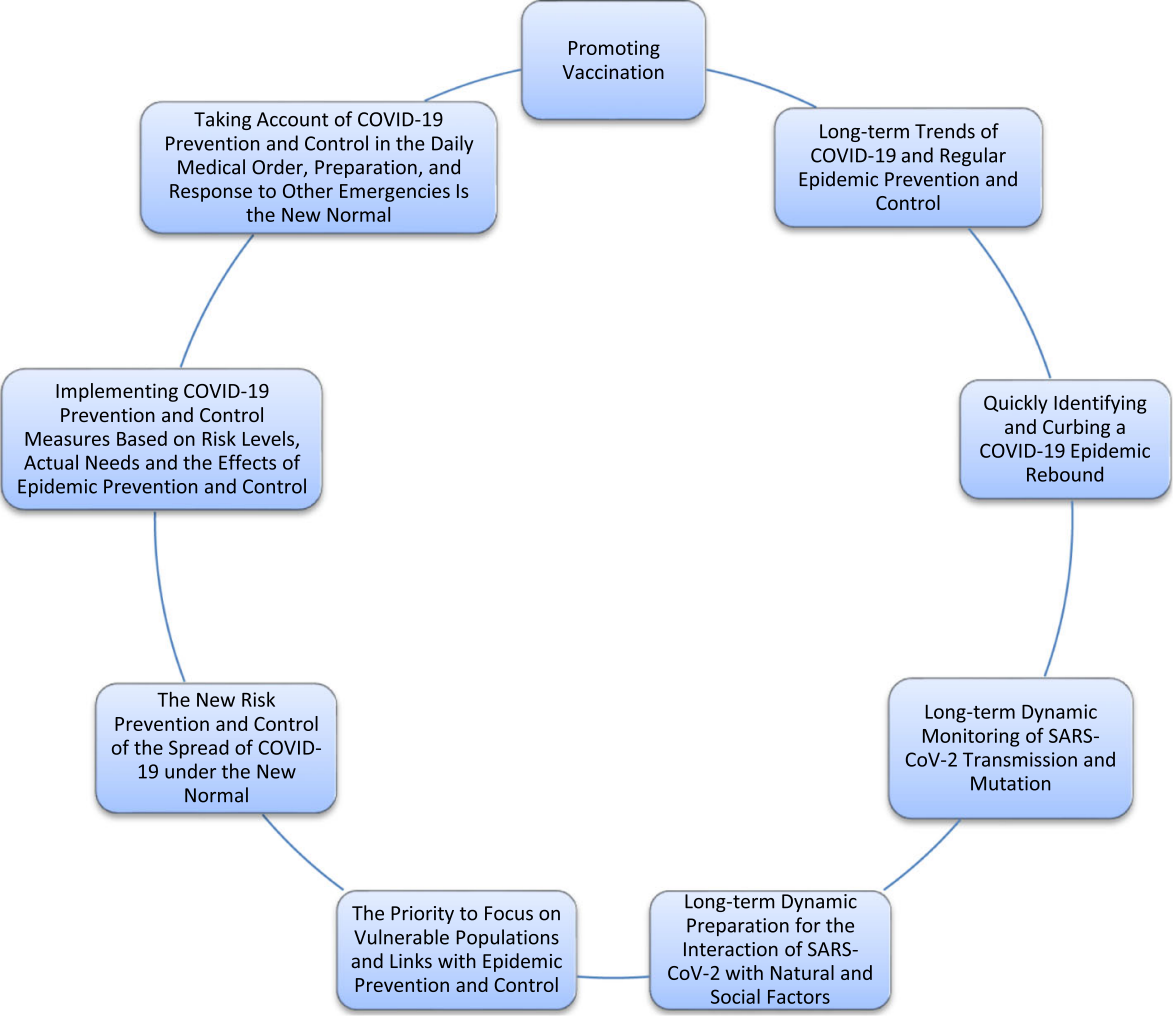
Dynamically adjusted strategy in response to developments in the COVID-19 pandemic as a new normal

The COVID-19 global pandemic is still ongoing, but epidemic trends and prevention and control effects in different countries and regions have obviously diverged. Compared with the initial stages, more epidemic prevention and control measures are currently available, but the complexity of the current epidemic presents larger and novel requirements for epidemic prevention and control. Therefore, in the current stage, rapid and precise epidemic prevention and control capabilities need to be further strengthened, as shown in Fig. 1. With the development of the situation of COVID-19 pandemic and SARS-CoV-2 mutation, the previous epidemic prevention and control measures, treatment measures and the protective effect of COVID-19 vaccine may change. Based on the close monitoring of the current COVID-19 situation and SARS-CoV-2 mutation, dynamic and targeted strategies are adopted. Therefore, dynamically adjusted strategies are more accurate compared to other strategies. In short, we must focus on making up for shortcomings and vulnerabilities in the prevention and control of COVID-19 in initial stages and pay attention to new vulnerabilities and new features that have emerged in the evolution of the COVID-19 epidemic. It is also necessary to make long-term preparations for the uncertainty and changes presented by COVID-19 in the future.

## Data Availability

All data generated or analyzed during this study are included in this manuscript.

## Abbreviations

COVID-19: Coronavirus disease 2019
SARS-CoV-2: Severe acute respiratory syndrome coronavirus 2
AIDS: Acquired immunodeficiency syndrome
